# High Fat Diet Feeding Exaggerates Perfluorooctanoic Acid-Induced Liver Injury in Mice via Modulating Multiple Metabolic Pathways

**DOI:** 10.1371/journal.pone.0061409

**Published:** 2013-04-23

**Authors:** Xiaobing Tan, Guoxiang Xie, Xiuhua Sun, Qiong Li, Wei Zhong, Peter Qiao, Xinguo Sun, Wei Jia, Zhanxiang Zhou

**Affiliations:** 1 Center for Translational Biomedical Research, University of North Carolina at Greensboro, North Carolina Research Campus, Kannapolis, North Carolina, United States of America; 2 Department of Nutrition, University of North Carolina at Greensboro, North Carolina Research Campus, Kannapolis, North Carolina, United States of America; 3 Department of Bioengineering, University of Pennsylvania, Philadelphia, Pennsylvania, United States of America; Indian Institute of Toxicology Reserach, India

## Abstract

High fat diet (HFD) is closely linked to a variety of health issues including fatty liver. Exposure to perfluorooctanoic acid (PFOA), a synthetic perfluorinated carboxylic acid, also causes liver injury. The present study investigated the possible interactions between high fat diet and PFOA in induction of liver injury. Mice were pair-fed a high-fat diet (HFD) or low fat control with or without PFOA administration at 5 mg/kg/day for 3 weeks. Exposure to PFOA alone caused elevated plasma alanine aminotransferase (ALT) and alkaline phosphatase (ALP) levels and increased liver weight along with reduced body weight and adipose tissue mass. HFD alone did not cause liver damage, but exaggerated PFOA-induced hepatotoxicity as indicated by higher plasma ALT and AST levels, and more severe pathological changes including hepatocyte hypertrophy, lipid droplet accumulation and necrosis as well as inflammatory cell infiltration. These additive effects of HFD on PFOA-induced hepatotoxicity correlated with metabolic disturbance in liver and blood as well as up-regulation of hepatic proinflammatory cytokine genes. Metabolomic analysis demonstrated that both serum and hepatic metabolite profiles of PFOA, HFD, or HFD-PFOA group were clearly differentiated from that of controls. PFOA affected more hepatic metabolites than HFD, but HFD showed positive interaction with PFOA on fatty acid metabolites including long chain fatty acids and acylcarnitines. Taken together, dietary high fat potentiates PFOA-induced hepatic lipid accumulation, inflammation and necrotic cell death by disturbing hepatic metabolism and inducing inflammation. This study demonstrated, for the first time, that HFD increases the risk of PFOA in induction of hepatotoxicity.

## Introduction

High fat diet (HFD) is a major cause of obesity which is closely linked to a variety of health issues, including coronary heart disease, stroke, high blood pressure, fatty liver disease, diabetes, and certain cancers [1]–[4]. HFD may contribute to the generation of free radical metabolites and the development of systemic inflammation and insulin resistance in these diseases [1], [2]. Recent studies have shown that high fatty acids in obesity may activate lipid-sense nuclear factors such as peroxisome proliferator-activated receptors (PPARs), liver X receptors, and farnesoid X receptors which play critical roles in cellular fatty acid and carbohydrate metabolism as well as cell proliferation [5], [6]. In addition to direct effects, dietary fat also plays an important role in modulating the metabolism and toxicity of environmental toxicants [7]. However, the extent and mechanisms of the interaction between fat and environmental toxicants have not been well investigated.

Perfluorooctanoic acid (PFOA) is a compound used extensively in industry and consumer products, and has been detected in the serum of most people in the United States with a median serum concentration of 4 ng/ml [8]. Individuals may be exposed to PFOA through drinking water and the consumption of foods prepared or packaged with Teflon containing products [9], [10]. In mammals, PFOA has been shown to be well absorbed orally. Upon absorption, PFOA accumulates preferentially in the liver and kidney, and is detectable in the bloodstream for an extended period of time, with a mean serum half-life of 4 years [11], [12]. Exposure to PFOA in rats and mice resulted in weight loss, liver injury, disorders of lipid metabolisms and thyroid functions [13], [14]. PFOA is structurally similar to fatty acids and has been found to bind PPAR-α, a key transcription factor in lipid metabolism [15]–[17]. In addition, epidemiological studies in human demonstrated that people with high serum PFOA levels have significantly elevated cholesterol and triglyceride levels [12]. This evidence suggests that the effects of PFOA occur as a result of the disturbance of lipid metabolism.

Liver injury is one of the major pathological consequences of dietary high fat intake and PFOA exposure. Although the impact of HFD and PFOA on liver metabolism remains unclear, a human study demonstrated that occupational exposure to PFOA elevated serum activities of liver enzymes only in obese subjects [18]. Due to the structural similarity of PFOA and fatty acids, HFD and PFOA may have synergistic effects on hepatic metabolic pathways. The present study utilized a mouse model to determine if a short-term HFD feeding exaggerates PFOA action on liver metabolism and organ injury.

## Materials and Methods

### Animals and PFOA Feeding Experiments

Male C57BL/6N mice were obtained from Charles River Laboratories (Wilmington, MA), and treated according to the experimental procedures approved by the North Carolina Research Campus Animal Care and Use Committee. PFOA was purchased from Sigma-Aldrich (St. Louis, MO). Mice at 4-month old were fed a liquid control diet or HFD [19] for 3 weeks with or without PFOA treatment. The calories concentration of either liquid control diet or HFD was 1,000 kcal/L. The calories compositions of the liquid control diet were 12% from fat, 18% from protein, and 60% from carbohydrate. The calories compositions of the liquid HFD were 35% from fat, 18% from protein, and 47% from carbohydrate. The dietary fat consisted of 71.7% olive oil, 21.5% corn oil and 6.8% safflower oil. All the gradients were purchase from Dyets (Bethlehem, PA). PFOA was prepared in deionized water at a concentration of 1 mg/ml in the stock solution, and mixed well in the liquid diet at a dosage of 5 mg/kg/day. To precisely control daily calories intake being the same for all the treatments, mice were pair-fed with the liquid diets. Because PFOA-fed mice showed the lowest food intake, PFOA-treated mice were fed *ad libitum*, and other groups were given the exact amount of diet consumed by the PFOA-treated mice in the previous day. At the end of feeding experiment, mice were anesthetized with inhalational isoflurane, and serum, liver and epididymal (eWAT) and subcutaneous white adipose tissues (sWAT) were collected.

### Hematology Parameters

Plasma alanine aminotransferase (ALT) and aspartate aminotransferase (AST) activities were determined using Infinity ALT and AST Reagents, respectively (Thermo Scientific, Middletown, VA). Blood glucose was measured using an OneTouch Ultra2 Blood Glucose Meter (Life Scan, Milpitas, CA). Plasma alkaline phosphatase (ALP), total and direct bilirubin, and free fatty acid concentrations (FFA) were determined with assay kits from BioVision (San Francisco, CA).

### Histopathology of Liver and eWAT and Hepatic Lipid Concentrations

Liver and eWAT tissues were fixed in 10% formalin and embedded in paraffin. Tissue sections were cut into 5 µm sections and processed for Hematoxylin & Eosin (H&E) staining. Hepatic lipids were extracted by homogenizing liver tissue in chloroform using 1% Trition X-100. The organic extracts were air dried, vacuumed, and dissolved in 1% Trition X-100. Triglyceride, cholesterol, and free fatty acid concentrations were determined using assay kits purchased from BioVision.

### qPCR

Total RNA was isolated from liver tissues with Trizol from Invitrogen (Carlsbad, CA), according to the manufacturer’s instructions. Reverse transcription was conducted with TaqMan Reverse Transcription Reagents (Applied Biosystems, Carlsbad, CA). qRT-PCR analysis was performed on Applied Biosystems 7500 Real Time PCR System (Applied Biosystems). Three mouse RT^2^ Profiler**™** PCR Arrays for fatty acid metabolism, glucose metabolism and drug metabolism, were purchased from SABioscience Corporation (Frederick, MD). Each RT^2^ Profiler**™** PCR Array monitors expression of 84 genes. For measurement of proinflammatory cytokine genes, primers (Table 1) were designed and synthesized by Integrated DNA Technologies (Coralville, IA), and qPCR were performed on the same systems as RT^2^ Profiler**™** PCR Arrays. For analysis of PCR Array data, the ΔΔCt method was used with the aid of a Microsoft excel spreadsheet containing algorithms provided by the manufacturer and each gene was normalized to β-actin. All the gene expression data were presented as relative expression (fold-change) between groups. A positive value indicates gene up-regulation and a negative value indicates gene down-regulation. Heat maps were generated using Gene Cluster 3.0 and Treeview software (http://rana.lbl.gov/EisenSoftware.htm).

**Table 1:** Primer sequences used for qPCR analysis.

Gene	Accession No.	Forward/Reverse (5′−3′)
KC/Cxcl1	NM_008176	AACCGAAGTCATAGCCACAC/CAGACGGTGCCATCAGAG
IL-1β/Il1b	NM_008361	ACGGACCCCAAAAGATGAAG/TTCTCCACAGCCACAATGAG
IP-10/Cxcl10	NM_021274	TCAGCACCATGAACCCAAG/CTATGGCCCTCATTCTCACTG
MCP-1/Ccl2	NM_011333	GTCCCTGTCATGCTTCTGG/GCTCTCCAGCCTACTCATTG
MIP-1α/Ccl3	NM_011337	GATTCCACGCCAATTCATCG/TTCAGTTCCAGGTCAGTGATG
MIP-1β/Ccl4	NM_013652	TGACCAAAAGAGGCAGACAG/GTGAGAAGCATCAGGGCTG
MIP-2/Cxcl2	NM_009140	GAAGTCATAGCCACTCTCAAGG/CTTCCGTTGAGGGACAGC
RANTES/Ccl5	NM_013653	GGGTACCATGAAGATCTCTGC/TCTAGGGAGAGGTAGGCAAAG
TNF-α/Tnf	NM_013693	CTTCTGTCTACTGAACTTCGGG/CAGGCTTGTCACTCGAATTTTG
β-Actin/Actb	NM_007393	GGCTGTATTCCCCTCCATCG/CCAGTTGGTAACAATGCCATGT

### Statistics

All data are presented as mean ± SD. The results were analyzed using Student’s *t*-test and One-Way ANOVA with Dunnett’s post hoc comparison for two groups and more than two groups, respectively. In all tests, *p* values less than 0.05 were considered statistically significant.

### Metabolomic Analysis

Serum and liver samples were prepared and analyzed with HPLC-TOF MS and GC-TOF MS as described in Dr. Wei Jia’s previous papers [20]–[23]. The ES+ and ES- raw data generated from HPLC-TOFMS was analyzed by MassHunter Qualitative Analysis Program (vB.05.01, Agilent, Santa Clara, CA) [21], [23]. The resulting data from the HPLC-TOFMS platforms were subjected to multivariate statistical analyses to establish characteristic metabolomic profiles associated with different groups. For GC-TOF MS generated data, the MS files were exported in NetCDF format by ChromaTOF software (v3.30, Leco Co., St. Joseph, Michigan). CDF files were extracted using custom scripts in MATLAB 7.1 (The MathWorks, Inc, Natick, MA) for data pretreatment. The resulting three dimension data set included sample information, peak retention time and peak intensities were be subjected to multivariate statistical analyses to establish characteristic metabolomic profiles associated with different experimental groups. The two data sets obtained from HPLC-TOFMS and GC-TOFMS were combined into a new data set and imported into SIMCA-P+12.0 software package (Umetrics, Umeå, Sweden). Principle component analysis (PCA) and orthogonal partial least squares-discriminant analysis (OPLS-DA) were carried out to visualize the metabolic alterations between each group. In addition to the multivariate statistical method, the Student’s *t*-test was also applied to determine the significance of each metabolite. Metabolites with both multivariate and univariate statistical significance (VIP>1 and *p*<0.05) are considered potential markers responsible for the differentiation of PFOA from controls or HFD-PFOA from PFOA group.

## Results

### Body and Liver Weights and Blood Parameters

As shown in Table 2, dietary PFOA exposure (5 mg/kg/day) for 3 weeks significantly increased liver weight and liver-to-body weight ratio, whereas the weights of eWAT and sWAT and total body weight were reduced. Although HFD alone did not affect liver weight and liver-to-body weight ratio, it exaggerated PFOA-induced increases in these parameters. Compared to the ALT activity (18.46 U/L) in controls, the plasma ALT activity in PFOA-treated mice was increased to 130.70 U/L, indicating a severe hepatic injury. HFD significantly enhanced PFOA-induced hepatotoxicity as indicated by a further increase in plasma ALT level (190.56 U/L) in mice exposed to both HFD and PFOA. However, elevation of plasma AST level was observed only in mice treated by HFD-PFOA. Plasma ALP level was elevated in both PFOA and HFD-PFOA groups with a greater value in PFOA group. There were no significant differences in plasma total and direct bilirubin levels among all the treatments. Plasma FFA level was not affected by either HFD or PFOA, but increased by co-exposure to HFD and PFOA.

**Table 2:** Body weights, organ weights and blood parameters in mice subjected to HFD or/and PFOA exposure for 3 weeks.

Measurements	CTRL	PFOA	HFD	HFD-PFOA
Body Weight (g)	26.38±0.84^a^	20.85±0.37^b^	28.93±0.58^c^	23.43±1.16^d^
Liver Weight (g)	1.02±0.04^a^	2.41±0.14^b^	1.15±0.13^a^	2.92±0.12^c^
eWAT Weight (g)	0.55±0.07^a^	0.12±0.04^b^	0.83±0.13^c^	0.31±0.19^b^
sWAT Weight (g)	0.36±0.06^a^	0.10±0.02^b^	0.50±0.04^c^	0.17±0.07^b^
Liver/BW (%)	3.89±0.07^a^	11.56±0.56^b^	3.98±0.45^a^	12.47±0.57^c^
eWAT/BW (%)	2.08±0.31^a^	0.59±0.20^b^	2.86±0.39^ac^	1.29±0.75^ab^
sWAT/BW (%)	1.38±0.21^a^	0.48±0.08^b^	1.74±0.15^c^	0.71±0.28^b^
Plasma ALT (U/L)	18.46±16.34^a^	130.70±37.11^b^	14.97±7.19^a^	190.56±64.64^c^
Plasma AST (U/L)	35.50±7.82^a^	57.26±13.20^ab^	35.12±3.74^a^	74.31±25.59^b^
Plasma ALP (U/dL)	0.06±0.01^a^	0.34±0.08^b^	0.04±0.01^a^	0.20±0.04^c^
Plasma total bilirubin (mg/dL)	2.04±1.13	1.90±1.21	1.58±1.24	1.44±0.59
Plasma direct bilirubin (mg/dL)	0.60±0.04	0.51±0.18	0.45±0.29	0.65±0.33
Plasma FFAs (mM)	2.49±0.91^a^	1.93±0.46^a^	2.41±0.45^a^	2.99±1.05^b^

Data are means ± SD (n = 7–8). Significant differences (*p*<0.05) among a, b, c, d were determined by One-Way ANOVA with Dunnett’s post hoc comparison. eWAT: Epididymal white adipose tissue. sWAT: Subcutaneous white adipose tissue. BW: Body weight. FFAs: Free fatty acids.

### Liver and WAT Histopathology

**Figure 1:**
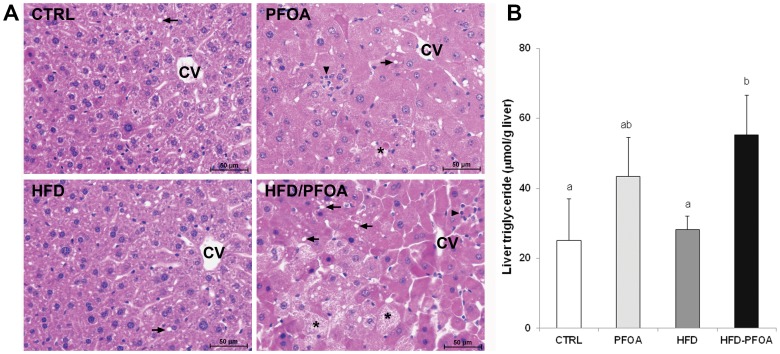
Histopathological alterations and triglyceride accumulation in the liver of mice fed HFD or/and PFOA. Adult mice were pair-fed a high-fat diet (HFD) or low fat control diet (CTRL) with or without perfluorooctanoic acid (PFOA) administration at 5 mg/kg/day for 3 weeks. A. Liver histopathology (H&E staining). Arrows: Lipid droplets. Arrowhead: Inflammatory cell infiltration. Asterisks: Necrosis. CV: Central vein. B. Liver triglyceride contents. Data are means ± SD (*n* = 7–8). Significant differences (*p*<0.05) between a and b were determined by One-Way ANOVA with Dunnett’s post hoc comparison.

**Figure 2:**
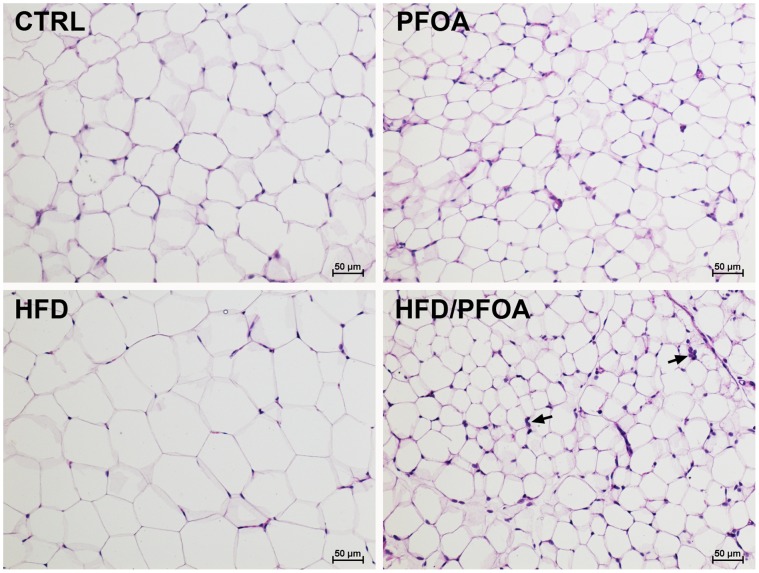
Histopathological alterations in the eWAT of mice fed HFD or/and PFOA. Adult mice were pair-fed a high-fat diet (HFD) or low fat control diet (CTRL) with or without perfluorooctanoic acid (PFOA) administration at 5 mg/kg/day for 3 weeks. H&E staining of Epididymal white adipose tissue (eWAT). Arrows: Inflammatory cell infiltration.

As shown in Figure 1A, PFOA exposure resulted in remarkable hepatocyte hypertrophy and degeneration as indicated by hepatocyte enlargement and lighter staining with eosin. PFOA also induced necrotic cell death as well as inflammatory cell infiltration. HFD alone did not cause significant histopathological changes in the liver. However, co-exposure to HFD and PFOA caused more severe liver damage compared to PFOA alone, as shown by greater levels of hepatocyte necrotic cell death and lipid droplet accumulation as well as inflammatory cell infiltration. Quantitative analysis of liver lipids showed a significant increase in hepatic triglyceride concentrations in HFD-PFOA group compared to control and HFD groups (Figure 1B). There were no significant differences in hepatic cholesterol and FFA concentrations among all the groups (Data not shown). In accordance with alterations of WAT mass as shown in Table 2, the adipocyte size was increased by HFD, but decreased by PFOA or HFD-PFOA treatment (Figure 2). Furthermore, inflammatory cell infiltration was observed in the WAT of HFD-PFOA group.

### Hepatic Genes Related to Fatty Acid Metabolism

**Figure 3:**
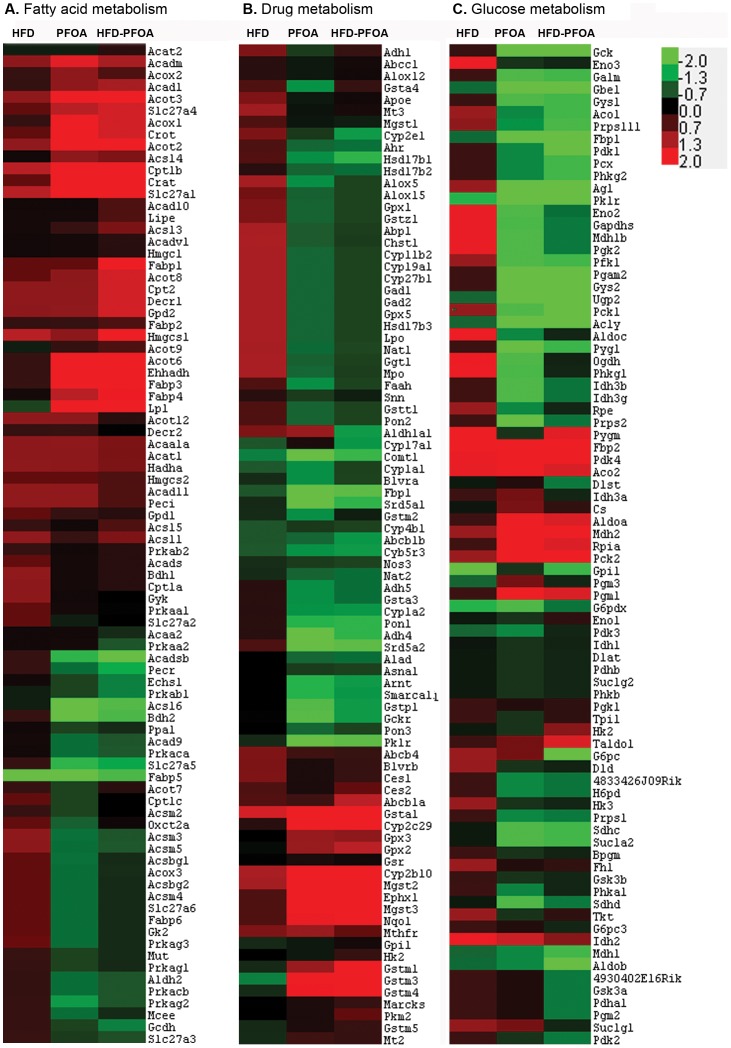
Gene expression profiles in the liver of mice fed HFD or/and PFOA. Adult mice were pair-fed a high-fat diet (HFD) or low fat control diet (CTRL) with or without perfluorooctanoic acid (PFOA) administration at 5 mg/kg/day for 3 weeks. The mRNA levels were analyzed by RT^2^ Profiler™ PCR Array (*n* = 3). **A**. Genes related to fatty acid metabolism. **B**. Genes related to drug metabolism. **C**. Genes related to glucose metabolism. Red and green corresponds to average up- and down-regulation, respectively.

Hepatic expression of 84 key genes involved in the regulation and enzymatic pathways of fatty acid metabolism was measured by RT^2^ Profiler™ PCR Array. As shown in Figure 3A, both HFD and PFOA showed significant effects on fatty acid metabolic genes related to all of the major pathways, including fatty acid catabolism, transport and triglyceride catabolism. Table 3 lists 33 fatty acid metabolic genes which were regulated ≥1.5 folds by HFD or/and PFOA. While HFD alone significantly up-regulated 8 genes, PFOA alone showed differential effects, i.e. up-regulated 13 genes (≥1.5 fold) and down-regulated 4 genes (≥1.5 fold). In particular, PFOA up-regulated Fabp3 and Slc27a1 by 112- and 28- folds, respectively. Among the 14 genes encoding mitochondrial and peroxisomal fatty acid oxidation enzymes tested, 12 genes were up-regulated by HFD or/and PFOA, and only Acadsb and Gcdh were down-regulated by PFOA and HFD-PFOA, respectively. HFD showed a negative interaction with PFOA in the regulation of most acyl-CoA oxidase genes, and positive interaction in most acyl-CoA synthesis/hydrolysis genes. While all the 5 acyl-CoA thioesterases were up-regulated by HFD, PFOA or HFD plus PFOA, the acyl-CoA synthetase was down-regulated. Among the 9 fatty acid transport genes, 8 genes were up-regulated and only Slc27a5 was down-regulated in PFOA groups. The triglyceride catabolism genes, Gpd2 and Lpl, were up-regulated by HFD and HFD-PFOA treatments.

**Table 3:** Fold changes of fatty acid metabolism genes in the liver of mice subjected to HFD or/and PFOA exposure for 3 weeks.

Genes	PFOA/CTRL	HFD/CTRL	HFD-PFOA/CTRL	HFD-PFOA/PFOA
**Acyl-CoA dehydrogenase**				
Acad10	1.1	1.1	1.6*	1.4*
Acad11	2.2*	2.2*	1.6*	−1.4*
Acadl	2.2*	1.4	2.5*	1.1
Acadm	3.5*	2.2	2.5*	−1.4
Acadsb	−2.9*	1.4*	−4.0*	−1.4*
Ehhadh	87.9*	1.4	100.6*	1.1*
Gcdh	−1.5	1.4*	−2.0*	−1.4*
**Acyl-CoA oxidase**				
Acox1	4.4*	1.4	3.2*	−1.4*
Acox2	2.2*	1.4	1.6*	−1.4
**Acyl-CoA thoesterases**				
Acot12	2.2*	2.2*	1.3	−1.7*
Acot2	112.0*	2.2	101.1*	−1.1*
Acot3	8.8*	2.2	12.7*	1.4*
Acot6	11.0*	1.4	12.6*	1.1*
Acot8	2.2*	1.7	3.2*	1.5*
**Acyl-CoA synthetases**				
Acsl6	−4.9*	−1.2	−3.4	1.4*
**Fatty Acid transport**				
Cpt1b	35.1*	2.8	40.3*	1.1*
Cpt2	2.2*	2.2*	3.2*	1.4*
Crat	7.0*	1.7	6.3*	−1.1*
Crot	4.4*	1.7	3.2*	−1.4*
Fabp1	1.7	1.7	4.0*	2.3*
Fabp3	111.8*	1.4	254.8*	2.3*
Slc27a1	27.7*	2.7	19.9*	−1.4*
Slc27a4	2.7	1.7	3.2*	1.2*
Slc27a5	−2.9*	1.4	−2.5*	1.2*
**Ketogenesis**				
Bdh2	−7.3*	1.4	−3.2*	2.3*
**Tricylglyceride catabolism**				
Gpd2	2.2*	2.2*	3.2*	1.4*
Lpl	13.9*	−1.5	12.7*	−1.1*
**Other**				
Hmgcs1	2.2	2.7*	5.0*	2.3*
Decr1	2.2*	2.2*	3.2*	1.4*
Echs1	−1.4	1.1	−2.0*	−1.4
Peci	2.2*	2.2*	1.6*	−1.4*
Pecr	−1.8*	1.4	−2.6*	−1.4*
Hprt1	2.2*	1.1	1.6*	−1.4*

The mRNA levels were analyzed by RT^2^ Profiler™ PCR Array (*n* = 3), and genes with ≥1.5 fold change are listed.

* Significant differences (*p*<0.05) were determined by Student’s *t*-test. CTRL: Control. HFD: High fat diet. HFD-PFOA: High fat diet plus PFOA.

### Hepatic Genes Related to Drug Metabolism

Hepatic gene expression of 84 drug metabolism genes was measured by RT^2^ Profiler™ PCR Array. Results are presented in Figure 3B in heat map and Table 4 in fold changes (larger than 1.5 fold). PFOA alone differentially altered hepatic expression of drug metabolism genes. The genes which were up-regulated more than 3-fold by PFOA included phase I genes, Cyp2b10 (12.5) and Cyp2c29 (11.6), and phase II genes, including Gstm3 (11.6), Gstm4 (3.7), Ephx1 (4.6), Nqo1 (3.6), Mgst2 (4.6) and Mgst3 (7.3). PFOA also down-regulated many genes related to drug metabolism; the genes, which were down-regulated more than 3-fold by PFOA, were phase II enzymes, including Adh4 (−7.0), Fbp1 (−11.2), Pklr (−5.5), Srd5a1 (−5.5), Srd5a2 (−8.8), Comt1 (−4.4) and Gckr (−3.5).

**Table 4:** Fold changes 0f drug metabolism genes in the liver of mice subjected to HFD or/and PFOA exposure for 3 weeks.

Genes	PFOA/CTRL	HF/CTRL	HFD-PFOA/CTRL	HFD-PFOA/PFOA
**Pgp**				
Abcb1a	1.5	1.6	2.8*	1.9
Abcb1b	−1.7*	−1.6*	−2.3*	−1.3
**P450 family**				
Cyp17a1	1.2	−1.6	−2.3	−2.6*
Cyp1a1	−2.2*	−1.6	−1.4*	1.5
Cyp2b10	12.5*	2.5	7.5*	−1.7
Cyp2c29	11.6*	1.3	11.0*	−1.1
Cyp2e1	−1.4	2.0*	−2.3	−1.7*
**Phase II Dehydrogenases**				
Adh4	−7.0*	1.3	−2.9*	2.4*
Hsd17b1	−2.17	1.6	−2.9*	−1.3
**Glutathione Peroxidase**				
Gpx2	2.3	−1	2.8*	1.2
Gpx3	2.3	−1	2.2*	−1.0
Gsta1	4.6*	3.2	14.0*	3.0*
Gstm1	2.3	−1.3	4.4*	1.9
Gstm3	11.6	−2	35.1*	3.0
Gstm4	3.7*	−1.3	5.5*	1.5
**Hydrolases**				
Ephx1	4.6*	1.6*	4.4*	−1.1
Fbp1	−11.2*	−1.6	−3.6*	3.1
**Kinases**				
Pklr	−5.5*	−1.3	−3.7*	1.5
**Oxidoreductases**				
Cyb5r3	−2.2*	−1.6*	−2.3*	−1.0
Gpx2	2.3	−1	2.8*	1.2
Nqo1	3.6*	1.6	4.4*	1.2
Srd5a1	−5.5*	−1.3	−2.9 *	1.9
Srd5a2	−8.8*	1.6	−3.7*	2.4*
**Paraoxonases**				
Pon1	−2.8*	1.3	−2.9*	−1.0
**Transferases**				
Comt1	−4.4*	−2.0*	−2.9*	1.5
**Glutathione S-Transferases**				
Gstm3	11.6	−2	35.1*	3.0
Gstm5	1.2	−1.3	1.4	1.2*
Mgst2	4.6*	2.5	4.4*	−1.1
Mgst3	7.3*	1.6	8.8*	1.2
**Others**				
Arnt	−2.8*	1	−2.3*	1.2
Gckr	−3.5*	−1	−2.3*	1.5
Smarcal1	−2.8*	1	−2.3*	1.2

The mRNA levels were analyzed by RT^2^ Profiler™ PCR Array (*n* = 3), and genes with ≥1.5 fold change are listed.

* Significant differences (*p*<0.05) were determined by Student’s *t*-test. CTRL: Control. HFD: High fat diet. HFD-PFOA: High fat diet plus PFOA.

HFD showed less effects on drug metabolism genes compared to PFOA. However, HFD showed remarkable interactions with PFOA in regulating hepatic drug metabolism genes. HFD had negative effects on PFOA-increased phase I gene expression, but had positive effects on PFOA-increased phase II gene expression. Co-exposure to HFD and PFOA further increased Gsta1, Gstm3 and Gstm4 by 3.0-, 3.0- and 1.5-fold, respectively, compared to PFOA alone. HFD also showed positive effects on PFOA-induced down-regulation of drug metabolism genes. All the genes down-regulated more than 3-fold by PFOA were attenuated more than 1.5-fold in HFD-PFOA group.

### Hepatic Genes Related to Glucose Metabolism

Hepatic expression of 84 key genes involved in glucose metabolism was measured by RT^2^ Profiler™ PCR Array, and the results are presented in Figure 3C in heatmap and Table 5 in fold changes (larger than 1.5 fold). Exposure to PFOA alone significantly down-regulated 5 genes related to glycolysis (Eno2, Gck), gluconeogenesis (Fbp1), TCA cycle (Sdha) and pentose phosphate (Prps2). In contrast, HFD significantly up-regulated 2 genes related to glycolysis (G6pc) and TCA cycle (Suclg1). HFD and PFOA showed remarkable interactions in modulating glucose metabolism genes, and co-exposure to HFD-PFOA significantly down-regulated 11 genes involved all the glucose metabolism pathways tested as compared to controls. While positive effects were found on 6 genes, HFD mainly showed negative effects over PFOA-modulated gene expression as indicated by significant lower expression of 14 genes in HFD-PFOA group than PFOA alone.

**Table 5:** Fold-change of glucose metabolism genes in the liver of mice subjected to HFD or/and PFOA exposure for 3 weeks.

Genes	PFOA/CTRL	HFD/CTRL	HFD-PFOA/CTRL	HFD-PFOA/PFOA	
***Glycolysis***					
Eno2	−1.8*	1.9	−1.4		1.4*
Gck	−11.6*	1.2	−7.0*		1.7*
Gpi1	−1.2	−2.1	−1.7		−1.5*
Hk2	−1.2	−1.1	1.5*		1.7*
Pgm2	1.1	1.2	−1.4*		−1.5*
Pgm3	1.4	−1.3	−1.1		−1.5*
Pklr	−9.2	−1.7	−5.5		1.7*
***Gluconeogenesis***					
Fbp1	−2.3*	−1.3	−3.5*		−1.5*
G6pc	1.4	1.5*	−2.8*		−3.8*
***Regulation***					
4930402E16Rik	1.1	1.2	−1.4*		−1.5*
Pdk1	−1.5	1.2	−2.2		−1.5*
***TCA cycle***					
Acly	−5.8	−1.3	−2.2		2.6*
Dlst	1.1	−1.1	−1.4*		−1.5*
Pdha1	1.1	1.2	−1.4		−1.5*
Sdha	−1.8*	−1.3	−2.8*		−1.5*
Suclg1	1.4	1.5*	−1.1		−1.5*
***Pentose Phosphate Pathway***					
Prps2	−2.3*	1.2	−1.4*		1.7*
Rbks	1.1	1.5	−1.4		−1.5*
***Degradation***					
Pgm2	1.1	1.2	−1.4*		−1.5*
Pgm3	1.4	−1.3	−1.1		−1.5*
***Regulation***					
Gsk3a	1.1	1.2	−1.4*		−1.5*

The mRNA levels were analyzed by RT^2^ Profiler™ PCR Array (*n* = 3), and genes with ≥1.5 fold change are listed.

* Significant differences (*p*<0.05) were determined by Student’s *t*-test. CTRL: Control. HFD: High fat diet. HFD-PFOA: High fat diet plus PFOA.

### Hepatic Genes Related to Cytokines

Hepatic expression of 9 major proinflammatory cytokine genes was assessed by real time RT-PCR, and the results are presented in Table 6. Exposure to PFOA significantly increased gene expression of MCP-1, MIP-1α and TNF-α with a fold change of 4.96, 4.85 and 2.49, respectively, when compared to the control. However, PFOA down-regulated Cxcl1(KC) expression. HFD alone did not cause any significant changes in hepatic expression of these proinflammatory cytokine genes. However, co-exposure to HFD and PFOA significantly up-regulated IP-10 (2.63-fold), MCP-1 (10.37-fold), MIP-1α (8.14-fold) and TNF-α (5.21-fold), compared to the control. HFD showed positive effects on PFOA-induced expression of proinflammatory cytokine genes as indicated by 2.11-, 2.09-, 1.68- and 2.11-fold increases in IP-10, MCP-1, MIP-1α and TNF-α, respectively, in the HFD-PFOA group when compared to PFOA alone.

**Table 6:** Fold changes of proinflammatory cytokine genes in the liver of mice subjected to HFD or/and PFOA exposure for 3 weeks.

Genes	PFOA/CTRL	HFD/CTRL	HFD-PFOA/CTRL	HFD-PFOA/PFOA	
KC	0.41*	1.04	0.47*	1.16	
IL-1b	1.28	1.00	1.10	0.85	
IP-10	1.25	0.93	2.63*	2.11*	
MCP-1	4.96*	1.62	10.37*	2.09*	
MIP-1a	4.85*	1.30	8.14*	1.68*	
MIP-1b	0.84	1.25	2.45	2.93	
MIP-2	0.82	1.04	1.02	1.25	
RANTES	1.36	0.96	0.91	0.67	
TNF-a	2.49	1.09	5.21*	2.11*	

The mRNA levels were analyzed by real time RT-PCR (*n* = 3, assay replicates = 4).

* Significant differences (*p*<0.05) were determined by Student’s *t*-test. CTRL: Control. HFD: High fat diet. HFD-PFOA: High fat diet plus PFOA.

### Plasma and Liver Metabolites

**Figure 4:**
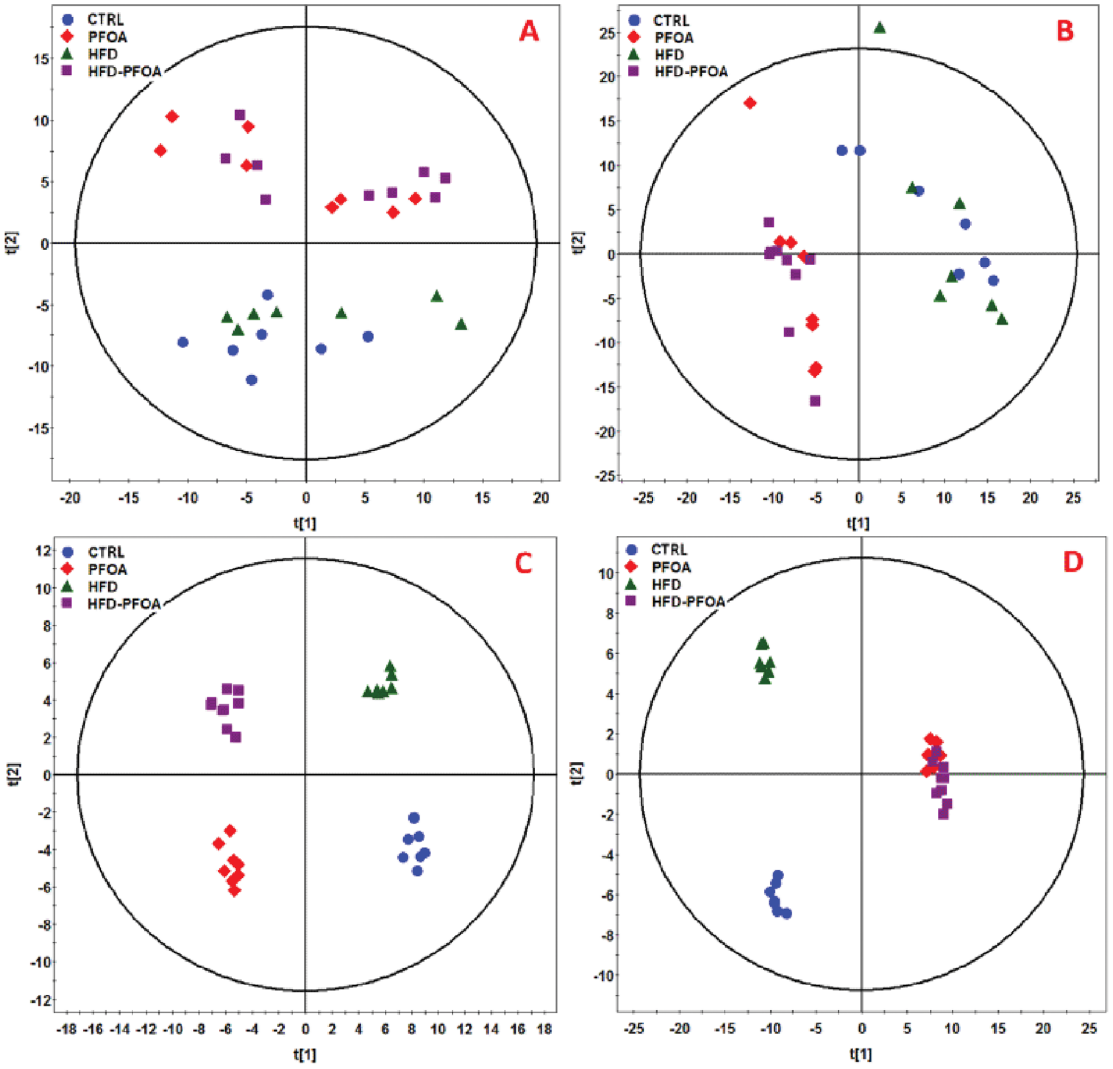
PCA and OPLS-DA scores plots of spectral data of serum and liver metabolites in mice fed HFD or/and PFOA. Adult mice were pair-fed a high-fat diet (HFD) or low fat control diet (CTRL) with or without perfluorooctanoic acid (PFOA) administration at 5 mg/kg/day for 3 weeks. Metabolite profiles of serum and liver tissue homogenates were analyzed by GC-TOFMS and HPLC-TOFMS (n = 7–8). **A**. PCA score plot of serum metabolites. **B**. OPLS-DA score plot of serum metabolites. **C**. PCA score plot of hepatic metabolites. **D**. OPLS-DA score plot of hepatic metabolites.

A total of 298 metabolites in plasma samples and 370 metabolites in liver samples were identified by LC-TOFMS and GC-TOFMS, of which 112 serum metabolites and 153 liver tissue metabolites were further validated by comparison to reference standards. According to the score plots of PCA and OPLS-DA model of subjects, the PFOA group, the HFD group, and the HFD plus PFOA groups were all clearly differentiated from controls (Figure 4). Moreover, the PFOA group was separated from the HFD plus PFOA group in serum samples but mixed in liver tissue samples.

Table 7 shows the list of differential serum and liver metabolites with a *p*<0.05. The altered serum metabolites included elevated palmitoleic acid and pantothenic acid, and decreased elaidic carnitine, trans-Hexadec-2-enoyl carnitine, cis-5-tetradecenoylcarnitine, palmitoylcarnitine, decosahexaenoic acid, and tetradecanoylcarnitine in PFOA treated mice compared to the controls. HFD alone increased serum arachidoic acid and linoleyl carnitine, but decreased serum long chain carnitines, including cis-5-tetradecenoylcarnitine, trans-Hexadec-2-enoyl carnitine and elaidic carnitine. Co-exposure to HFD and PFOA reduced more serum metabolites compared to PFOA or HFD alone, and the interactions of HFD and PFOA on serum metabolites were mostly negative as indicated by the fold changes of HFD-PFOA over PFOA.

**Table 7:** Fold changes of serum metabolites in mice subjected to HFD or/and PFOA exposure for 3 weeks.

Compounds	FC^a^ (PFOA/CTRL)	p^b^ (PFOA/CTRL)	FC^a^ (HFD/CTRL)	p^b^ (HFD/CTRL)	FC^a^ (HFD-PFOA/CTRL)		p^b^ (HFD-PFOA/CTRL)	FC^a^ (HFD-PFOA/PFOA)	p^b^ (HFD-PFOA/PFOA)
**Cystathionine**	0.58	2.73E-09	0.89	1.68E-02		0.54	8.51E-09	0.93	3.23E-01
**Glucose**	0.47	3.34E-05	0.83	7.45E-02		0.40	5.14E-07	0.85	2.50E-01
**Arabinofuranose**	0.49	9.88E-05	0.78	2.26E-02		0.39	3.30E-06	0.80	1.46E-01
**Palmitoylcarnitine**	0.56	1.37E-04	0.78	8.03E-02		0.45	3.13E-06	0.79	5.13E-02
**Stearoylcarnitine**	0.63	1.68E-04	1.09	4.93E-01		0.69	8.48E-04	1.08	3.76E-01
**cis-5-Tetradecenoylcarnitine**	0.64	2.91E-04	0.61	1.13E-03		0.53	4.29E-05	0.82	1.49E-01
**trans-Hexadec-2-enoyl carnitine**	0.69	5.86E-04	0.45	1.94E-05		0.40	2.10E-07	0.58	1.52E-04
**Arginine**	0.59	7.55E-04	0.84	1.66E-01		0.38	6.65E-07	0.65	2.63E-03
**Glucuronic acid**	3.74	7.94E-04	1.04	8.90E-01		2.76	4.13E-05	0.74	8.82E-02
**Glycerolphosphate**	0.59	7.99E-04	0.90	2.69E-01		0.63	3.85E-04	1.07	4.68E-01
**Palmitoleic acid 1**	1.91	1.63E-03	0.62	9.32E-02		2.04	2.45E-04	1.07	4.21E-01
**Propionylcarnitine**	2.61	3.10E-03	0.95	7.91E-01		2.14	6.77E-04	0.82	2.74E-01
**Glycerol**	0.64	3.63E-03	0.92	4.37E-01		0.81	2.16E-01	1.27	2.12E-01
**Elaidic carnitine**	0.69	4.14E-03	0.64	4.66E-03		0.55	3.75E-04	0.79	1.94E-01
**Galacose**	0.77	7.20E-03	0.97	8.66E-01		0.85	1.40E-02	1.10	2.99E-01
**Tetradecanoylcarnitine**	0.38	1.22E-02	0.53	7.44E-02		0.26	6.33E-04	0.68	3.25E-01
**Cholesterol**	1.61	1.31E-02	0.97	7.35E-01		1.56	2.12E-04	0.97	8.13E-01
**Arachidonic acid**	0.44	1.41E-02	1.74	5.06E-03		0.64	4.46E-02	1.45	1.96E-02
**Acetylcarnitine**	1.39	1.52E-02	0.87	4.19E-01		1.26	1.71E-01	0.91	4.94E-01
**Glutamate**	1.31	1.66E-02	0.83	1.71E-01		1.02	8.12E-01	0.78	9.32E-03
**Decosahexaenoic acid**	0.55	1.80E-02	1.41	7.71E-02		0.50	9.06E-03	0.92	7.28E-01
**Norophthalmic acid**	1.37	1.87E-02	0.87	2.20E-01		1.21	2.53E-01	0.88	3.79E-01
**Fructose 1,6-bisphosphate**	1.31	2.66E-02	1.01	9.23E-01		1.09	3.58E-01	0.83	7.03E-02
**Pantothenic acid**	2.27	3.76E-02	0.99	9.31E-01		2.28	1.49E-02	1.01	9.84E-01
**Linoleyl carnitine**	1.10	6.51E-01	2.58	1.09E-04		2.26	1.08E-05	2.05	1.71E-04
**Lysine**	1.18	3.04E-01	1.27	1.67E-01		0.72	5.58E-02	0.61	3.04E-03
**Trimethylamine N-Oxide**	1.46	1.07E-01	0.67	1.96E-01		0.97	8.82E-01	0.66	7.65E-03
**Glutathione**	1.03	8.83E-01	1.30	3.70E-01		0.56	7.66E-03	0.54	1.63E-02
**Ornithine**	0.99	8.47E-01	0.84	2.28E-02		0.83	1.24E-02	0.84	2.06E-02
**Lactate**	1.08	6.35E-01	0.71	1.81E-01		0.82	2.77E-01	0.75	2.86E-02
**Cysteineglutathione disulfide**	1.14	4.82E-01	1.15	5.46E-01		0.70	6.38E-02	0.61	3.03E-02

Serum metabolites were analyzed by GC-TOF MS and HPLC-TOFMS (n = 7–8).

a Fold change (FC) was obtained by comparing those metabolites in perfluorooctanoic acid (PFOA) group, high fat diet (HFD) group and HFD-PFOA group to control group (CTRL) or HFD-PFOA group to PFOA group; FC with a value >1 indicates a relatively higher concentration present in PFOA group, HFD group or HFD-PFOA group while a value <1 means a relatively lower concentration as compared to the controls or a relatively higher concentration present in HFD-PFOA group while a value <1 means a relatively lower concentration as compared to the PFOA group.

b *p* values from Student’s *t*-test.

Table 8 shows the list of differential liver metabolites with a *p*<0.05. PFOA exposure remarkably increased the levels of fatty acids including linoleyl carnitine, malonylcarnitine, decosahexaenoic acid, palmitoleic acid, eicosatrienoic acid, palmitoylcarnitine, octadecanoic acid, and 11, 14-eicosadienoic acid. In contrast, the levels of glucose, galactose, ribose, mannitol and fructose were all dramatically reduced by PFOA exposure. The major effects of HFD were increases in linoleyl carnitine, decosahexaenoic acid, galactosylglycerol and linoleic acid, although it also decreased citric acid. Co-exposure to HFD and PFOA showed more significant effects than HFD or PFOA alone. HFD potentiated the variations of the differential metabolites upon PFOA exposure, and the most significant interactions between HFD and PFOA were on S-(hydroxymethyl) glutathione, linoleic acid and gamma-linoleic acid as indicated by 3-, 2.51- and 1.84-fold increases in HFD-PFOA group compared to PFOA alone.

**Table 8:** Fold changes of hepatic metabolites in mice fed HFD or/and PFOA exposure for 3 weeks.

Compounds	FC^a^ (PFOA/CTRL)	p^b^ (PFOA/CTRL)	FC^a^ (HFD/CTRL)	p^b^ (HFD/CTRL)	FC^a^ (HFD-PFOA/CTRL)	p^b^ (HFD-PFOA/CTRL)	FC^a^ (HFD-PFOA/PFOA)	p^b^ (HFD-PFOA/PFOA)
**Linoleyl carnitine**	12.24	2.14E-02	3.30	2.30E-02	19.63	1.99E-03	1.60	2.08E-01
**3-Phosphoglyceric acid**	7.57	1.29E-03	1.36	3.31E-02	5.12	1.71E-05	0.68	1.31E-01
**Decosahexaenoic acid**	5.56	2.30E-04	6.40	7.42E-03	6.99	2.45E-06	1.26	1.76E-01
**trans-Hexadec-2-enoyl carnitine**	5.29	7.36E-03	0.51	5.65E-02	3.45	2.17E-03	0.65	1.65E-01
**Elaidic carnitine**	4.75	3.79E-02	0.66	2.05E-01	3.66	2.97E-02	0.77	5.13E-01
**Pantothenic acid**	4.66	4.84E-07	1.29	2.64E-01	4.78	9.34E-08	1.03	7.82E-01
**Palmitoleic acid 1**	4.57	4.17E-03	0.90	6.24E-01	5.56	4.95E-06	1.22	3.16E-01
**Malonylcarnitine**	4.44	9.49E-04	0.98	9.76E-01	7.37	1.11E-05	1.66	1.31E-02
**Eicosatrienoic acid**	4.07	2.68E-05	1.10	7.77E-01	5.03	6.71E-08	1.24	7.59E-02
**Palmitoylcarnitine**	3.42	4.56E-02	1.10	9.68E-01	3.70	1.58E-02	1.08	8.19E-01
**Carnitine**	3.22	3.16E-06	1.11	7.15E-01	2.99	1.18E-06	0.93	4.53E-01
**Glycerolphosphate**	2.59	5.28E-05	1.51	1.61E-02	3.02	7.08E-04	1.17	3.68E-01
**Lysine**	2.32	1.50E-02	1.70	1.12E-01	2.10	1.46E-02	0.91	6.62E-01
**3-Hydroxybutyric acid**	2.31	8.62E-03	1.12	3.47E-01	3.33	1.14E-01	1.44	4.40E-01
**Octadecanoic acid, 2,3-bishydroxypropyl ester**	1.97	1.10E-02	1.74	2.15E-01	2.16	4.71E-03	1.10	5.12E-01
**Galactosylglycerol**	1.90	5.04E-03	4.17	2.49E-02	2.31	2.61E-02	1.21	3.86E-01
**Glyceric acid**	1.82	1.09E-03	1.18	1.15E-01	1.47	1.86E-02	0.80	1.08E-01
**Spermidine**	1.81	2.83E-04	1.04	5.35E-01	1.60	9.91E-04	0.88	1.80E-01
**Threonic acid**	1.66	4.88E-04	1.16	9.39E-02	1.65	5.65E-02	1.00	9.86E-01
**2-Glycerolphosphate**	1.62	8.08E-05	1.27	2.83E-02	1.69	2.00E-03	1.04	6.90E-01
**11,14-Eicosadienoic acid**	1.51	4.81E-02	1.34	1.87E-02	2.08	1.81E-03	1.37	9.47E-02
**Choline**	1.45	2.56E-02	1.17	1.36E-01	1.55	9.79E-03	1.07	6.45E-01
**Cysteine**	1.44	1.35E-02	1.37	2.35E-01	1.50	1.94E-02	1.04	7.64E-01
**Glutamine**	1.21	4.25E-03	1.08	2.55E-01	1.11	1.97E-01	0.92	1.78E-01
**Glucose**	0.81	1.42E-02	1.05	7.77E-01	0.67	4.43E-03	0.82	4.45E-02
**Phosphocholine**	0.71	3.63E-02	0.87	4.37E-01	0.61	1.74E-03	0.85	2.74E-01
**Dodecanoic acid**	0.70	1.43E-02	1.01	9.82E-01	0.58	1.14E-03	0.84	1.53E-01
**Glyceraldehyde**	0.62	9.50E-03	1.26	3.79E-01	0.64	4.48E-03	1.03	8.16E-01
**Arachidic acid**	0.56	1.01E-02	1.38	2.13E-02	0.54	5.83E-04	0.96	8.29E-01
**Citric acid**	0.55	1.95E-02	0.42	6.75E-03	0.68	3.58E-01	1.23	6.55E-01
**S-(Hydroxymethyl) glutathione**	0.54	4.59E-03	0.90	7.53E-01	1.62	1.78E-01	3.00	2.03E-02
**Myristic acid**	0.45	2.87E-02	0.67	1.84E-01	0.36	1.49E-02	0.80	5.08E-02
**Propionylcarnitine**	0.41	6.45E-03	1.06	7.41E-01	0.56	1.71E-02	1.38	4.13E-01
**Glutamic acid**	0.39	4.47E-04	1.50	1.93E-02	0.39	5.24E-05	1.00	9.98E-01
**Ribitol**	0.34	1.00E-07	0.92	5.40E-01	0.35	7.30E-10	1.00	9.75E-01
**Malic acid**	0.31	2.97E-03	1.60	2.12E-02	0.34	7.20E-04	1.10	6.60E-01
**Fumaric acid**	0.31	5.93E-03	1.54	4.99E-02	0.36	3.52E-03	1.17	4.12E-01
**Galactose**	0.30	6.95E-06	0.86	5.91E-01	0.22	2.36E-08	0.74	2.26E-01
**Ribose**	0.30	4.72E-05	1.38	4.69E-02	0.36	2.81E-05	1.20	2.71E-01
**Mannitol**	0.28	4.01E-07	1.06	6.41E-01	0.21	7.27E-08	0.76	1.34E-01
**3-Methylglutarylcarnitine**	0.25	3.11E-04	0.78	3.29E-01	0.37	7.06E-04	1.47	3.03E-01
**Fructose**	0.12	1.33E-05	0.80	5.78E-01	0.08	5.15E-07	0.69	1.31E-01
**Linoleic acid**	1.58	2.78E-01	4.96	1.89E-02	3.97	3.95E-05	2.51	3.18E-04
**Gamma-Linoleic acid**	0.49	4.20E-01	0.99	8.61E-01	0.90	8.89E-01	1.84	1.95E-03
**Lactate**	0.94	4.02E-01	1.06	7.54E-01	0.75	4.62E-03	0.80	3.43E-02

Hepatic metabolites were analyzed by GC-TOF MS and HPLC-TOFMS (n = 7–8).

a Fold change (FC) was obtained by comparing those metabolites in perfluorooctanoic acid (PFOA) group, high fat diet (HFD) group and HFD-PFOA group to control group (CTRL) or HFD-PFOA group to PFOA group; FC with a value >1 indicates a relatively higher concentration present in PFOA group, HFD group or HFD-PFOA group while a value <1 means a relatively lower concentration as compared to the controls or a relatively higher concentration present in HFD-PFOA group while a value <1 means a relatively lower concentration as compared to the PFOA group.

b *p* values from Student’s *t*-test.

## Discussion

The health risk of PFOA exposure has come under increased scrutiny during the last decade due to the finding that PFOA is persistent in the environment and was detected in significant levels in the blood of animals as well as human beings [8], [24]. Structurally similar to fatty acids, PFOA has been shown to induce peroxisome proliferation, disrupt homeostasis of fatty acids and cholesterol metabolism, and leads to fatty liver [13]–[17]. PFOA exerts its toxic effects mainly through activation of PPARα, which is also a key nuclear factor regulated by natural and endogenous fatty acids. HFD has been closely linked to many health issues including obesity, coronary disease, stroke, high blood pressure, diabetes and certain cancers. However, little is known about the influence of high fat diet on PFOA induced-liver toxicity. In the present study, HFD potentiated PFOA-induced liver toxicity as indicated by significant lipid accumulation, increased inflammation, hepatocyte necrosis, and metabolism disturbances, even though HFD along did not cause significant changes in the liver due to diet restriction in pair-feeding to a lower diet intake observed in PFOA groups.

Numerous animal studies have demonstrated that PFOA induced lipid accumulation in the liver. In addition to reducing secretion of very low density lipoprotein (VLDL) from the liver [25], PFOA may also increase release from peripheral adipose tissues and/or lipid uptake by hepatocyte via up-regulated CD36 protein [25]–[27]. Corroborating these findings, the present study showed that PFOA exposure resulted in microvesicular fatty liver as well as peripheral adipose atrophy. The loss of adipose mass may mechanistically link with the gain of liver lipids as we recently documented a reverse triglyceride transport from adipose tissue to the liver due to hyper-lipolysis in the adipose tissues after alcohol exposure [28]. High fat diet alone increased body weight as well as peripheral adipose tissues, but did not significantly affect liver weight and lipid contents. However, HFD had additive effects on PFOA-induced liver weight gain and liver-to-body weight ratio. Although HFD did not significantly affect PFOA-induced loss of adipose tissue mass, PFOA-induced liver weight gain and hepatic lipid accumulation was exaggerated by HFD. Because HFD dramatically increased hepatic linoleic acid level (4.96-fold increase vs. control), an increased supply of fatty acids to the liver may contribute to the positive interaction of HFD with PFOA in induction of hepatic lipid accumulation in the present study.

PPAR α is a major transcription factor responsible for regulation of fatty acid metabolism in peroxisomes, mitochondria, and smooth endoplasmic reticulum in the hepatocyte [29]–[31]. Certain fatty acids serve as activator of PPARα in regulation of lipid metabolism [32], [33]. In the present study, HFD elevated expression of fatty acid metabolism genes related to fatty acid β-oxidation and fatty acid transport, which are mainly regulated by PPARα. Possibly due to the structural similarities of PFOA and fatty acids, PFOA appears to be a potential activator of PPARα in several animal studies. In PFOA-treated mice, major gene expression changes were demonstrated to be PPAR α-dependent because alterations occurred in wild type mice but not in PPARα null mice [15], [34]. In addition, wild type mice exposed to PFOA presented a pathological and histological pattern related to effects of PPARα activators, which did not occur in PPARα null mice [17], [34]. Consistent with these findings, PFOA treated animals in the present study markedly up-regulated gene expression associated with mitochondrial and peroxisomal fatty acid β-oxidation and fatty acid transport including Cpt1, Acox1 and Fabp3. Addition of HFD further elevated expression of these genes, especially for fatty acid β-oxidation genes, Ehhadh, Acot3, Acot8, Cpt1a and Cpt2, and fatty acid transport genes, Fabp1 and Fabp3. These results support the idea that HFD might produce additive effects in PFOA-induced liver toxicity via action on PPARα transcription factor.

Activation of PPARα pathway may lead to increased fatty acid uptake and oxidation, reduced glucose utilization, and the accumulation of lipid intermediate metabolites in hepatocytes [31], [35]. In the presence of PFOA, the levels of most fatty acid metabolites were elevated in the liver and glucose levels were suppressed in both serum and liver, corroborating to PPARα activation in liver. The most remarkable effects of HFD and PFOA on hepatic metabolites are the increases in long chain fatty acids such as linoleic acid and long chain acylcarnitine such as linoleyl carnitine. HFD and PFOA showed synergistic effects on hepatic fatty acid metabolites, especially the long chain acylcarnitines, including linoleylcarnitine, malonylcarnitine and eicosatrienoic acid. The increases in hepatic long chain fatty acids and acylcarnitines indicate a disorder of fatty acid oxidation. Hepatic accumulation of fatty acids may cause cell injury by directly inducing lipotoxicity or indirectly generating toxic byproducts such as caramide and lysophosphatidyl choline [36], [37]. Fatty acids might be able to activate toll-like receptor-4, destabilize lysosomal membranes, or promote release of cathepsin B, leading to activation of apoptotic pathways [37]–[40]. In addition, altered cellular lipid metabolism could lead to lipotoxicity in liver through activation of protein kinase c and ceramide-mediated cellular injury [36], [41]. The data of hepatic metabolite profiling in the present study clearly demonstrated disorders in multiple pathways involved in fatty acid metabolism, and provide us a clue for future mechanistic investigations on HFD-PFOA interactions in induction of liver injury.

Body weight loss and hypoglycemia was associated with PFOA-induced hepatic lipid accumulation and liver injury regardless of HFD in the present study. Metabolomics analysis demonstrated that PFOA not only affects fatty acid metabolism, but also interferes with other metabolic pathways, particularly glucose metabolism, in the liver. In addition to glucose, PFOA exposure significantly reduced the levels of a battery of metabolites including fructose, mannitol, galactose, fumaric acid, malic acid and citric acid. The reduction of these metabolites correlated well with the down-regulation of several glucose metabolism genes. Surprisingly, HFD also significantly reduced hepatic citric acid level by 0.42-fold decrease compared to the control. Depletion of hepatic citric acid by PFOA or HFD indicates mitochondrial dysfunction in tricarboxylic acid (TCA) cycle. Although a large number of fatty acid metabolism genes were up-regulated by PFOA or HFD, the increase in fatty acids/fatty acylcartinines and decrease in glucose metabolites in the present study suggest that PFOA or HFD exposure causes an incomplete mitochondrial fatty acid β-oxidation and a poor energy generation. Down-regulation of genes involved in TCA cycle, including Acly, Dlst, Pdha, Sdha and Suclg1, by PFOA and HFD further supports the metabolomics observation on the defect of mitochondrial TCA cycle. Taken together, induction of hepatic disorders in fatty acid and glucose metabolism are major molecular mechanisms underlying the deleterious effects of PFOA and HFD on liver function.

We also found that the expressions of the proinflammatory cytokine genes, TNF-α, MCP-1, MIP-1α, IP-10α, were greater in the liver of HFD-PFOA group than PFOA alone, suggesting that HFD exacerbated the inflammation in the PFOA-exposed mice. PFOA has been shown in animal studies to modulate immune functions by altering T-lymphocyte response and induce inflammation by increasing expression of proinflammatory cytokines such as IL-6, TNF-α, IL-1 β [42]–[44]. Oxidative stress induced by PFOA on the liver in fatty acid oxidation promoted expression of proinflammatory cytokines in animals [43], [45]. In the present experiment, HFD may amplify these effects through further disturbance of lipid metabolism in liver and caused higher levels of proinflammatory cytokines in PFOA exposed mice with HFD. These proinflammatory cytokines exert pathophysioligical effects in the liver as they mediate inflammation and tissue injury [46].

In conclusion, HFD and PFOA exposure synergistically induces liver injury, including lipid accumulation, inflammation and necrotic cell death. Gene expression and metabolomics analysis showed that both HFD and PFOA significantly disturb hepatic metabolism in association with activation of PPARα. This study demonstrated for the first time that HFD increases the risk of PFOA exposure in induction of liver injury.
